# Ovarian dysgerminoma with pseudo-Meigs syndrome: A case report

**DOI:** 10.1097/MD.0000000000026319

**Published:** 2021-06-11

**Authors:** Xuebo Li, Deqing Chen, Xiuhui Jin, Guangtao Xu, Bo Hu, Xiansi Zeng, Xin Jin

**Affiliations:** a Key Laboratory of Evidence Identification in Universities of Shandong Province, Shandong University of Political Science and Law, Jinan, SD; b Forensic and Pathology Laboratory, Jiaxing University Medical College, Jiaxing, ZJ, China; c Department of Immunology and Human Biology, University of Toronto, Toronto, ON, Canada; d Department of Pathology and Key-Innovative Discipline Molecular Diagnostics, Jiaxing Hospital of Traditional Chinese Medicine, Jiaxing University, Jiaxing, ZJ, China.

**Keywords:** dysgerminoma, ovary, pseudo-Meigs syndrome

## Abstract

**Rationale:**

Dysgerminoma is a rare malignant tumor of the ovary, more frequently occurring in young women. The main signs of pseudo-Meigs syndrome (PMS) are ascites and hydrothorax accompanying benign or malignant ovarian tumors (no fibroma or fibroma-like tumor).

**Patient concerns:**

A 19-year-old woman with fever and chest tightness for 2 days.

**Diagnoses:**

Pectoral-abdominal computed tomography (CT) scan and contrast-enhanced magnetic resonance imaging revealed a large amount of right pleural effusion, a small amount of ascites, and a huge abdominopelvic mass measuring about 29.2cm × 11.8cm × 8.4 cm in the left ovary. The result of hydrothorax examination was consistent with the diagnosis of exudative pleural effusion. In addition, Rivalta-test showed a positive result and lactate dehydrogenase was elevated. The histopathological diagnosis was a giant germ cell tumor, which was consistent with dysgerminoma in terms of both morphology and immunophenotype. Based on these findings, a diagnosis of malignant ovarian neoplasm with PMS was made.

**Interventions:**

Surgical resection of the tumor was performed.

**Outcomes:**

The patient recovered well after operation, and the pleural effusion and abdominal ascites vanished. No recurrence was observed during the 1-year follow-up period.

**Lessons:**

Ovarian dysgerminoma with PMS is a rare malignant tumor of the ovary, which often occurs in young women. It should be considered in differential diagnosis of patients with a pelvic mass, ascites and pleural effusion. Early diagnosis and surgical treatment are beneficial to prolonged survival.

## 1 Introduction

Ovarian germ cell tumors originate from primordial germ cells, accounting for 20% to 25% of all ovarian neoplasms, but only 3% to 5% of them are malignant.^[1]^ As a malignant ovarian germ cell tumor, dysgerminoma is rare, accounting for 2% of all ovarian tumors. About 75% dysgerminomas occur in adolescents and young adults.^[2]^ Dysgenic gonads and sexual maldevelopment as represented by Turner syndrome, testicular feminization and triple X syndrome are supposed to be related to the occurrence of dysgerminoma.^[3]^

Meigs syndrome is a rare condition that manifests as an ovarian fibroma or fibroma-like tumor with ascites and pleural effusion. The hydrops usually resolves after removal of the tumor.^[4]^ After that, another syndrome of the same type may appear, which is termed as pseudo-Meigs syndrome (PMS). But whether the tumor type in PMS is benign or malignant was not initially described by Meigs despite the similar clinical presentation; it may be a mature teratoma, struma ovarii, ovarian carcinoma, or metastatic gastrointestinal malignancy.^[5]^

Here we report a case of ovarian dysgerminoma complicated by PMS, which is extremely rare in the literature. It is our hope that this case report could enrich the information about the clinical manifestations of this disease.

## 2 Case presentation

A 19-year-old women who complained of fever and chest tightness for 2 days presented to the emergency room of our hospital on March 11, 2020. An urgent chest computed tomography (CT) scan as part of the workup for possible pulmonary disease revealed a large amount of right pleural effusion and a small amount of left pleural effusion, for which a drainage tube was indwelled to expel the pleural effusion. Hydrothorax examination showed a positive Rivalta test result and elevation of lactate dehydrogenase (LDH), which was consistent with exudative pleural effusion, and the diagnosis of a malignant tumor was suspected. However, cytological examination did not find malignant cells in the pleural fluid.

**Figure 1:**
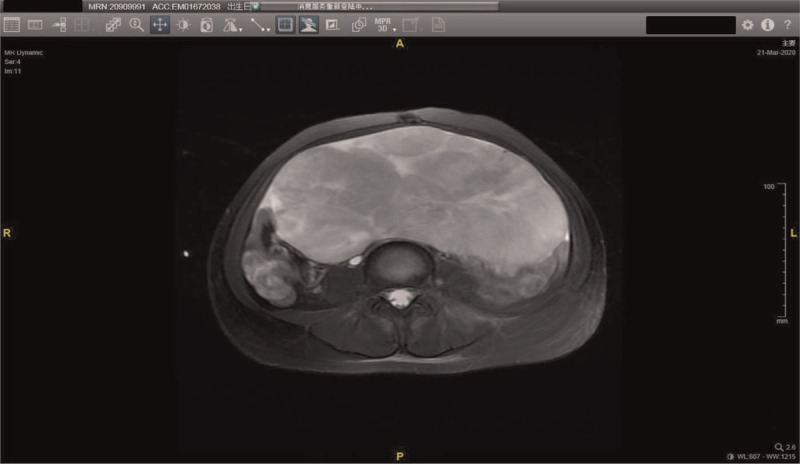
Contrast-enhanced MRI shows a huge abdominopelvic mass.

Abdominal and pelvic CT scans revealed a huge abdominopelvic mass measuring about 29.2 cm × 11.8 cm × 8.4 cm, with a small amount of ascites. The tumor also compressed the right ureter, causing mild hydronephrosis. Subsequent contrast-enhanced magnetic resonance imaging (MRI) revealed the mass with solid and cystic components on the left side of the abdominopelvic cavity, which was suspected as an ovarian neoplasms (Fig. 1). Serological assessment showed that LDH, carbohydrate antigen 125 (CA-125), neuron-specific enolase and risk of ovarian malignancy algorithm (ROMA) were all elevated as 11,794 U/L (normal 135–215 U/L), 410.70 U/mL (normal < 35 U/mL), > 500.00 ng/mL (normal < 15 ng/mL), and 27.1% (normal < 7.4%), respectively. In addition, serum hormone levels were negative. The patient was diagnosed with ovarian malignancy.

After perfecting all related preoperative examinations, left oophorosalpingectomy with radical lymphatic nodes dissection was performed, revealing a tumor measuring about 29.0 cm × 20.0 cm × 13.0 cm in the left ovary, which was diagnosed as a malignant tumor by rapid frozen intraoperative pathology, with no peripheral tissue infiltration and lymph node metastasis. No abnormality was seen in the right ovary.

**Figure 2:**
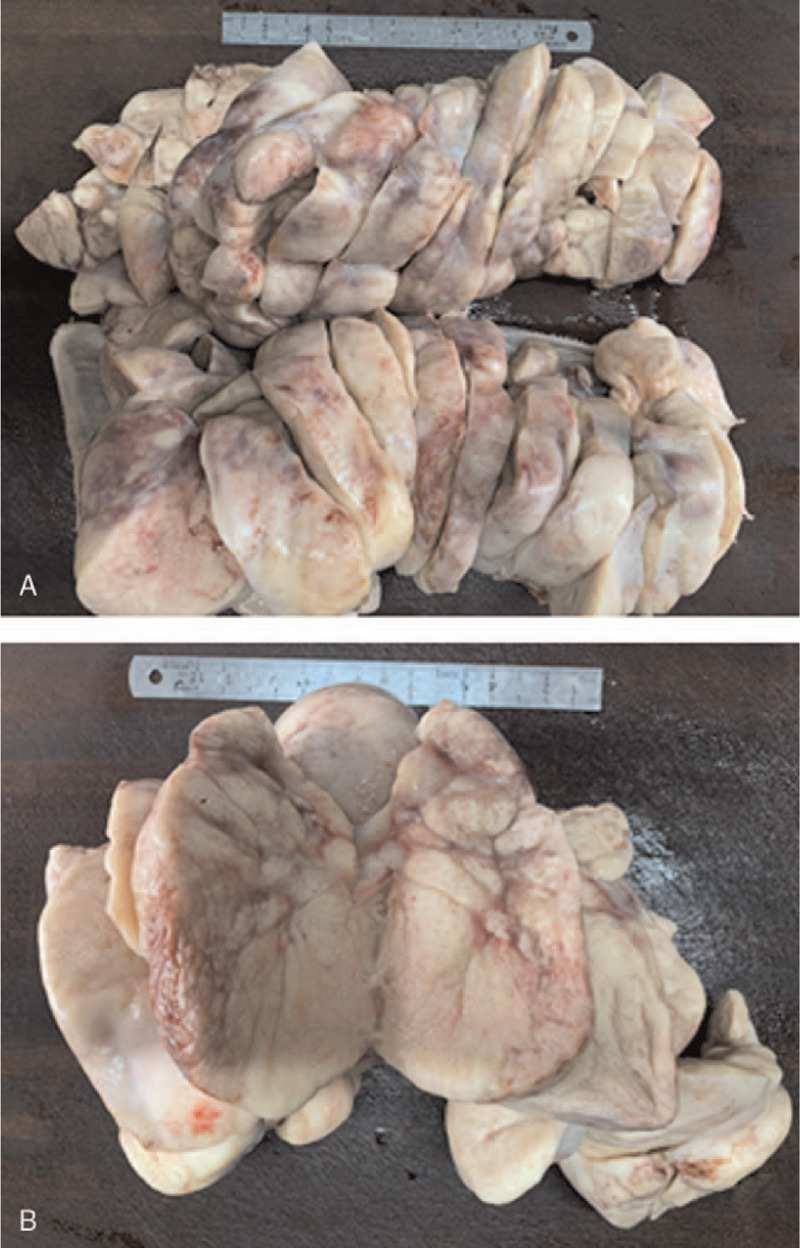
The tumor presents a solid, flesh and cream-colored cross section with no obvious necrosis.

**Figure 3:**
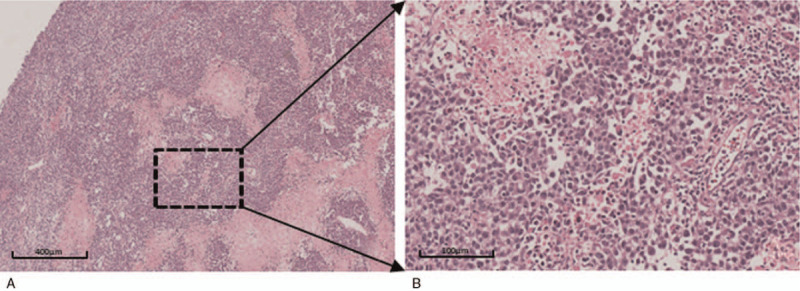
(A) Tumor cells are arranged in a nest-like and slab-like pattern. (B) Diffusely arranged polygonal cells with lymphocytes (HE staining: A, × 50; B, × 200).

Routine histopathology showed that the tumor was solid, flesh and cream-colored without obvious necrosis (Fig. 2). The tumor tissue was composed of polygonal cells with clear cytoplasm, large roundish nuclei and prominent nucleoli. The cells showed a nest-like and slab-like pattern arrangement with visible mitoses, cataplasia, vacuolation, and hemorrhagic necrosis. The parenchymal cells were separated by strands of fibrous tissue. Lymphocyte infiltration was observed in the stroma (Fig. 3). There was no pathological evidence of metastasis of the tumor to the left fallopian tube.

**Figure 4:**
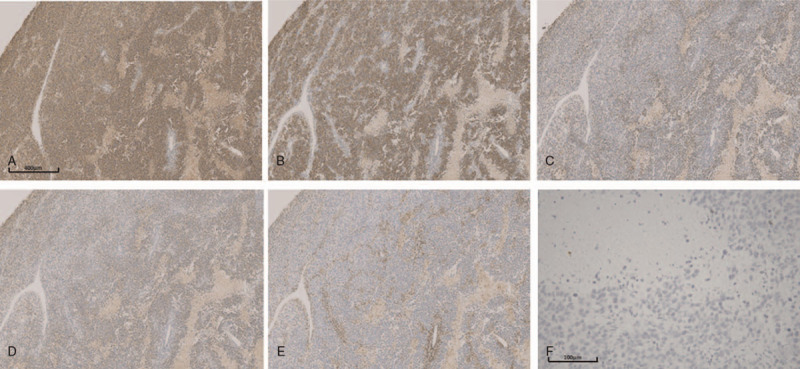
Immunoperoxidase staining indicates D2–40 + (A, × 50), CD117 + (B, × 50), CK (focal areas, +) (C, × 50), placental alkaline phosphatase+ (D, × 50), leukocyte common (lymphocyte, +) (E, × 50) and HCG (individual cell, +) (F, × 200) in the tumor mass.

Immunohistochemical results were as follows: creatine kinase (focal areas, +), vimentin (−), leukocyte common antigen (lymphocyte, +), placental alkaline phosphatase (+), cluster differentiation 30 (CD30) (−), CD117 (+), α-fetoprotein (−), podoplanin (+), human chorionic gonadotropin (HCG) (individual cell, +), and inhibin-α (−) (Fig. 4). The final pathological diagnosis was a giant germ cell tumor, which was consistent with dysgerminoma in both morphology and immunophenotype.

The patient recovered well postoperatively and received no further treatment. The pleural and peritoneal effusions resolved completely a week after surgery. One month after operation, LDH, CA-125, NSE, and ROMA levels returned to the normal ranges. The patient has been followed up for a year, and no sign of recurrence has been observed so far.

## 3 Discussion

According to previous reports, the incidence of dysgerminoma is relatively low, and most cases occurred in women younger than 20 years. Fortunately, ovarian dysgerminoma can usually be detected in the early stage and has a good prognosis after systemic therapy, with an overall survival rate of more than 80%. Surgery is often a curative therapy for early-stage ovarian dysgerminoma. The cure rate of a unilateral tumor without capsular invasion or spread is high as 96%.^[6]^ Dysgerminomas typically demonstrates a reasonable response rate to chemotherapy and radiation therapy. Some patients can receive bleomycin, etoposide, and cisplatin according to the surgical method and tumor stage.^[7]^ The classical clinical manifestations of dysgerminoma are an abdominal mass and pressure symptoms with elevation of serum LDH.^[8,9]^ The presence of tumor nest multiplicity, central blood vessels and ovarian vascular pedicle on radiological images may aid in diagnosing dysgerminoma.^[10]^ This tumor is the ovarian counterpart of testicular seminoma and is therefore histologically similar. About 80% to 90% dysgerminomas are unilateral and consist of undifferentiated germ cells and large vesicle cells scattered in the bed sheet or umbilical cord.^[11]^ Immunohistochemistry often showed positive CD117 in about 80% cases, which may be correlated with the presence of a *KIT* gene mutation.^[12]^ The positive expression of organic cation/carnitine transporter 4, spalt like transcription factor 4 and D2–40 may also be detected in dysgerminoma.^[1,13]^ Although dysgerminoma does not always affect hormone secretion, positive HCG expression of syncytiotrophoblast giant cells was observed in 3% cases. As in other benign or malignant ovarian neoplasms, dysgerminoma may contain functional ovarian stroma with luteinized stromal cells.^[6]^ In the present case, the histopathological examination results were consistent with the diagnosis of dysgerminoma; and the absence of malignant cells in the pleural fluid meant no pleural metastasis of malignant tumors.

An ovarian mass, ascites, pleural effusion, and elevated CA-125 almost indicate the presence of malignant disease and/or metastasis. In 1887, Demons et al reported the first case of pleural effusion associated with a benign ovarian cyst.^[14]^ Until 1937, this syndrome was named Meigs syndrome when Meigs and Cass presented a case series of patients who had ascites and hydrothorax in association with an ovarian fibroma.^[15]^ Subsequently, some scholars have reported several cases of benign and malignant ovarian tumors (no fibroma or fibroma-like tumor) with pleural effusion and ascites. They termed this clinical manifestation as PMS.^[16,17]^ Until now, the pathophysiology of these syndromes remains unclear. Researchers find that the pleural effusions in most patients with this entity tumor are inflammatory exudates, which may be related to the translocation of ascites via diaphragmatic pores, the release of inflammatory cytokines and growth factor, and the obstruction of venous and/or lymphatic drainage by a pelvic tumor.^[18]^ A definitive diagnosis of Meigs syndrome or PMS can only be made in retrospect following the resolution of fluid accumulations after removing the ovarian tumor.^[19]^ It is of clinical significance to use these syndromes as the differential diagnosis of pleural effusion, especially in young women who desire fertility or pregnant.

In summary, we have presented a rare case of ovarian dysgerminoma with PMS. Ovarian dysgerminoma should be considered in young women with a pelvic mass, ascites, and pleural effusion.

## Author contributions

**Conceptualization:** Xin Jin.

**Investigation:** Guangtao Xu.

**Supervision:** Xin Jin.

**Writing – original draft:** Xin Jin, Xuebo Li, Deqing Chen, Xiuhui Jin, Guangtao Xu, Bo Hu, Xiansi Zeng.

**Writing – review & editing:** Xin Jin, Xuebo Li, Guangtao Xu.
